# Compound Heterozygous Variants in the Phospholipase Gene PNPLA6 Cause Hypopituitarism and Vision Loss

**DOI:** 10.1155/humu/4515038

**Published:** 2026-06-19

**Authors:** Sebastian Vishnopolska, James Liu, Maria Andrea Camilletti, Julian Martinez Mayer, Lucia Iglesias Garcia, Michelle Brinkmeier, Elisa Vaiani, Sofia Hebe Vidal, Marta Ciaccio, María Isabel Di Palma, Alicia Belgorosky, Marcelo Marti, Robert B. Hufnagel, Sally A. Camper, Maria Ines Perez-Millan

**Affiliations:** ^1^ Department of Human Genetics, University of Michigan Medical School, Ann Arbor, Michigan, USA, umich.edu; ^2^ Ophthalmic Genetics and Visual Function Branch, National Eye Institute, National Institutes of Health, Bethesda, Maryland, USA, nih.gov; ^3^ Institute of Neurosciences (INEU-CONICET), Foundation for the Fight Against Neurological Diseases of Childhood (FLENI), Buenos Aires, Argentina; ^4^ Institute of Physiology, Molecular Biology and Neurosciences (IFIBYNE) CONICET, University of Buenos Aires, Buenos Aires, Argentina, uba.ar; ^5^ Endocrinology Service-CONICET, Garrahan Hospital, Buenos Aires, Argentina; ^6^ Ophthalmology Service, Garrahan Hospital, Buenos Aires, Argentina; ^7^ Department of Biological Chemistry, Faculty of Exact and Natural Sciences, University of Buenos Aires (FCEyN-UBA) and Institute of Biological Chemistry of the Faculty of Exact and Natural Sciences (IQUIBICEN) CONICET, Buenos Aires, Argentina

**Keywords:** Boucher–Neuhäuser syndrome, Gordon–Holmes syndrome, growth hormone deficiency, Laurence–Moon syndrome, neuropathy target esterase, Oliver–McFarlane syndrome, retinitis pigmentosa, spastic paraplegia type 39

## Abstract

PNPLA6 is a conserved lysophospholipase essential for maintaining nervous system integrity. Biallelic mutations in *PNPLA6* have been identified in individuals with a broad spectrum of disorders that can include ataxia, vision loss, and pituitary hormone deficiency. Here, we report the identification of novel compound heterozygous variants in *PNPLA6* (p.T1115P and p.Pro1142_Ala1143ins14) in a 10‐year‐old girl with combined pituitary hormone deficiency, including growth hormone, thyroid‐stimulating hormone, and gonadotropins. She also has vision loss and neurodevelopmental delay. Functional validation demonstrates that both variants, a missense substitution affecting a highly conserved residue within the catalytic domain and an intronic variant generating a novel splice acceptor site, completely abolish NTE activity, establishing their pathogenicity. Little is known about the cause of hypopituitarism in individuals with *PNPLA6* deficiency. Here, we report the cell‐type‐specific expression of PNPLA6 in mouse pituitary development and in adult animals. PNPLA6 is expressed broadly in SOX2+ stem cells within the pituitary primordium as early as e10.5, prior to lineage specification, suggesting a role in progenitor maintenance and early differentiation. In neonates and adults, expression predominates in the cells that produce growth hormone and pro‐opiomelanocortin. These findings suggest that PNPLA6 could influence pituitary development at early stages, as well as contribute to the altered function of hormone‐secreting cells.

## 1. Introduction

The *PNPLA6* gene encodes the neuropathy target esterase (NTE) enzyme, a highly conserved lysophospholipase anchored to the cytoplasmic face of the endoplasmic reticulum (ER). The main function of this enzyme is to deacylate phosphatidylcholine and lysophosphatidylcholine to glycerophosphorylcholine and maintain the homeostasis of ER phospholipids [1, 2]. Reduced NTE enzymatic activity leads to variable neurodegenerative symptoms, sometimes associated with spastic paraplegia, ataxia, neuropathy, and retinal dystrophy. Some patients have pituitary hormone deficiencies [3].

Pathogenic variants in *PNPLA6* have been reported in several clinical syndromes that range in severity. Oliver–McFarlane syndrome (MIM 275400) is a rare congenital disorder characterized by severe chorioretinal atrophy that can lead to childhood blindness, trichomegaly, and multiple pituitary hormone deficiencies that can include growth hormone (GH), gonadotropins, and thyroid‐stimulating hormone (TSH). Congenital hypogonadism occurs in half of the patients [1]. Laurence–Moon syndrome (MIM 245800) includes all the symptoms of the Oliver–McFarlane syndrome plus neurological symptoms, such as ataxia, spastic paraplegia, and neuropathy [4]. Finally, Gordon–Holmes (MIM 212840) and Boucher–Neuhäuser (MIM 215470) include cerebellar ataxia and hypogonadotropic hypogonadism, and the latter also includes chorioretinal dystrophy [5].

Recent studies have suggested that pathogenic variants in PNPLA6 can cause a wide spectrum of phenotypes because missense alleles impair the activity of the enzyme to different degrees [3]. Retinopathy and endocrinopathy tend to be associated with more severe impairment. To better understand the mechanism of PNPLA6 function in the nervous system, studies have been carried out in invertebrate and vertebrate loss of function models. The Swiss cheese protein (sws) is the *Drosophila* orthologue of vertebrate PNPLA6, and it is involved in survival and reproductive success. Neuronal‐specific knockdown of sws causes reduced longevity, locomotor and memory deficits, mitochondrial abnormalities, lipid droplet accumulation, and severe, progressive, brain neurodegeneration [6]. In the retina, PNPLA6 is mainly expressed in retinal pigment epithelial (RPE) cells and contributes to the mobilization of choline. Retina‐specific depletion of PNPLA6 in mice led to retinitis pigmentosa that can be rescued by choline supplementation [7]. Some of these features mimic those identified in humans with loss‐of‐function mutations. In mice, *Pnpla6* is expressed in the brain, pituitary, retina, lens, testis, and kidney. *Pnpla6* deficiency is lethal in mice, and brain‐specific mutants exhibit neurodegeneration [6]. However, little is known about the cell‐specific expression pattern and role of NTE during pituitary gland development and the mechanism by which PNPLA6 deficiency causes hypopituitarism.

In this study, we identified novel compound heterozygous *PNPLA6* variants in a patient with pituitary hormone deficiency and vision loss. We performed functional assays to document the pathogenicity and quantify the loss of function caused by these variants. We also report the cell‐type‐specific expression of PNPLA6 in mouse pituitary development and in adult animals.

## 2. Methods

### 2.1. Clinical Evaluations

The human subject research study was approved by the ethical committee of the Hospital Garrahan, Buenos Aires, Argentina, in accordance with the Declaration of Helsinki (Protocol Number 1828). Adult individuals or the parents of children signed a written informed consent to participate.

Patients were diagnosed with hormone deficiencies according to previously described and internationally accepted criteria [8, 9]. The serum levels of sex hormones, thyroid hormone, GH, and IGF‐1 were tested. The proband was examined by ultrasound, magnetic resonance imaging (MRI) of the brain, the hypothalamic–pituitary area, and the pelvis, and a hand X‐ray to assess bone age by Greulich and Pyle bone age atlas. A detailed ophthalmic examination, including visual acuity, visual field, fundoscopy, and optical coherence tomography (OCT), was also conducted.

### 2.2. Genetic Studies

Genomic DNA was extracted from peripheral blood samples using the Puregene Blood kit (Qiagen, Hilden, Germany) according to the protocol provided by the manufacturer. The concentration and purity of DNA were detected by a NanoDrop 2000 and QuantiFluor dsDNA System (Promega, Madison, Wisconsin, United States). DNA concentration was normalized to 25 ng/*μ*L for the panel assay and 20 ng/*μ*L for whole genome sequencing. The absorbance ratio 260/280 nm was used to assess the purity of DNA. A ratio between 1.8 and 2.1 was used as an inclusion criterion for DNA sample processing in the following steps.

The patient described here was initially analyzed using a custom gene panel that used single‐molecule molecular inversion probe sequencing (smMIPS) to detect genetic variants (for details, see our previous publication) [9]. This panel captures exonic regions of 67 hypopituitarism genes.

Whole genome sequencing was performed on genomic DNA from blood samples from the proband and her parents. Sequencing was performed on NovaSeq (S4) 300 cycles at Novagen.

Sanger sequencing was performed to validate genomic variants and monitor segregation within the family. Primers were designed around the mutations using Primer 3 software and synthesized by IDT (Integrated DNA Technologies, United States). Genomic DNA was used as a template and amplified by PCR using Taq Polymerase (HighWay). Sequencing was done at Macrogen, Inc., and results were analyzed with FinchTV software.

### 2.3. Bioinformatics

FASTQ raw reads were filtered by quality and aligned to GRCh38 using the Burrows‐Wheeler Aligner (BWA) [10] and processed according to the best practice recommendations from the Broad Institute‐Genome Analysis Toolkit (GATK) [11]. The resulting VCF was then annotated using dbSNP, ExAC, gnomAD, ClinVar, Polyphen, SIFT, MutationTaster, ACMG automated classification, and SpliceAI predictions. Annotated VCF files were uploaded to Bitgenia’s platform (https://www.bitgenia.com/b-platform/). Genetic variants were screened using the panel of 51 candidate genes, filtered by population frequency (< 0.01 in gnomAD), model of inheritance, and SpliceAI predictions of splice disruptive variants (maxSAI ≥ 0.1).

For structural modelling, the wild‐type structure was downloaded from UniProt (AF‐Q8IY17‐F1) (no crystal structure is currently available). The mutated structures were determined using Alphafold2 through the online Galaxy platform, by modifying the corresponding amino acids [12] [13]. The mutant structures were aligned to the wild‐type structures independently using the VMD Multiset function and then visualized using VMD [12].

### 2.4. *PNPLA6* Transcript Analysis

RNA was prepared from peripheral blood with RNAqueous Total RNA Isolation Kit (Thermo Fisher) and quantified by 260 nm absorbance. First‐strand cDNA was prepared by SuperScript II (Invitrogen) using oligo‐dT as primer. Primers were designed to amplify from Exon 29 to Exon 31 of the *PNPLA6* gene. The forward primer was 5 ^′^CACCTACTCATGGATGGCGG3 ^′^, and the reverse primer was 5 ^′^GCCGGTCTGTGAGCATTTTC3 ^′^. PCR amplification was carried out under the following conditions: 98°C 2 min, {98°C 30 s, 60°C 30 s, 68°C 30 s} × 35, 68°C × 5 min using a Pfu DNA polymerase (PBL). PCR products were separated on a 1.5% agarose gel. The reference allele produces a 416 bp product, and the splice variant allele produces a 458 bp product.

### 2.5. Protein Expression and Sample Preparation

PNPLA6 constructs were expressed and prepared using the methods described [3, 14]. Briefly, full‐length *PNPLA6* cDNA (from *RefSeq* transcript NM_001166111.2) was subcloned into a pcDNA6/V5‐His B plasmid. p.T1115P, c.3428‐44G>A, and p.R1031Qfs∗38 constructs were generated by mutagenesis and sequenced verified by LifeSCT (Rockville, MD). Plasmids were transfected into HEK293 suspension cells using polyethylenimine (PEI) at a 4:1 PEI:DNA mass ratio.

### 2.6. NTE Enzymatic Assay

NTE enzymatic activity assay was performed by a previously described colorimetric assay [15, 16]. NTE activity was defined as the difference in phenyl valerate hydrolysis activity inhibited by paraoxon (inhibits background esterase activity) and paraoxon + mipafox (inhibits NTE in addition to background esterase activity). Endpoint absorbance was measured at 486 nm using a Synergy 2 microplate reader (Biotek, Winooski, Vermont, United States). Specific NTE protein concentration was determined by SDS PAGE and Coomassie using previously defined methods [17]. Li‐Cor Odyssey DLx and Image Studio Lite (Li‐Cor, Lincoln, Nebraska, United States) was used to image and quantify the intensity of the bands. Band intensity was converted to a protein concentration using a bovine serum albumin (BSA) standard curve ranging from 0.25 to 1 mg/mL on the same gel. Final protein concentration was determined by taking the average concentration of at least three independent samples. We define one unit of NTE‐specific activity as 1 mmol phenol produced per minute per milligram protein.

### 2.7. Immunostaining

Embryos, heads from P0 neonates, and adult pituitaries (6 weeks) were fixed for 2–4 h in 4% formaldehyde in PBS (pH 7.2) at room temperature, dehydrated in a graded series of ethanol, and embedded in paraffin. Six micrometer thick sections were prepared for immunohistochemistry. Paraffin sections were incubated in sodium citrate buffer 10 mM, pH 8.5 at 80°C for 10 min to retrieve the antigens. Incubation with primary antibodies was performed overnight at 4°C. Sections were washed twice in PBS for 20 min at room temperature and incubated 1 h at room temperature with biotinylated secondary antibodies. After washing twice in PBS, sections were incubated with the tyramide signal amplification (TSA) fluorescein isothiocyanate (CF488 or CF543) kit (according to the manufacturer’s protocol, Perkin‐Elmer, Boston, MA). All sections were incubated with DAPI to stain cell nuclei. Finally, they were mounted in ProlonGold (Life Technologies). All images were taken using an Olympus FluoView 500 Laser Scanning Confocal Microscope.

Immunostaining for pituitary hormone markers was performed using antibodies for TSH*β* (RRID:AB_2756856), GH (RRID:AB_2665564), ACTH (RRID:AB_2802130), LH*β* (RRID:AB_3591788), and PRL (RRID:AB_2629220) from the National Hormone and Peptide Program (UCLA Medical Center, Torrance, California, United States) at 1:1000 dilution. Immunostaining for PNPLA6 and SOX2 was performed using a rabbit polyclonal anti‐PNPLA6 (1:100) from Abcam (ab228683) and goat polyclonal anti‐SOX2 (1:100) from R&D Systems (RRID:AB_355110).

## 3. Results

### 3.1. Clinical Characteristics

The proband was a 10‐year‐old female (46, karyotype) seen at the Garrahan Hospital in Buenos Aires, Argentina, born to nonconsanguineous healthy parents by cesarean section. She was small for gestational age (SGA), with a birth weight of 1800 g (−2.46 SDS) and a birth length of 42 cm (−2.94 SDS). She was discharged without neonatal complications and subsequently showed recovery of postnatal growth.

**Figure 1:**
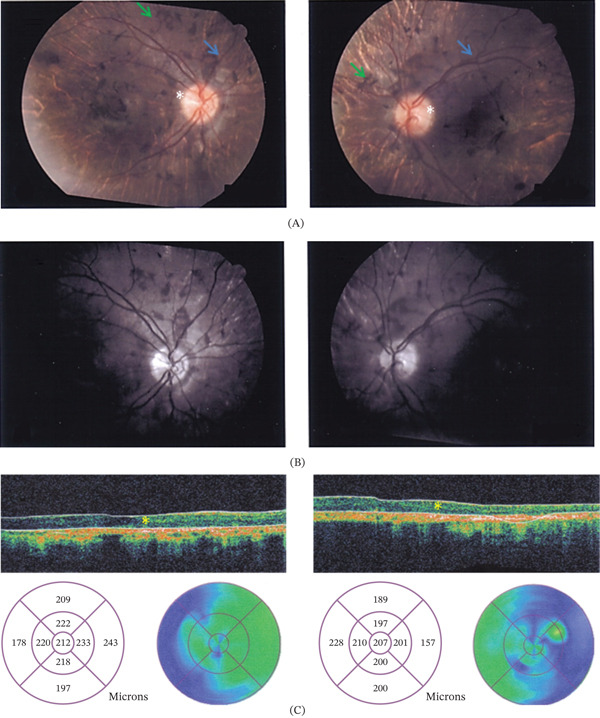
Clinical features of an individual with hypopituitarism and visual impairment. (A) Color retinography of the right and left eye shows temporal pallor of the optic nerve (white asterisk), macula with scattered pigment clumps, vascular thinning (blue arrows), and pigment deposits in pattern of bone spicules (green arrows). The left and right columns are the left and right eyes. (B) Fundus auto fluorescence highlights pigment deposits as hypoautofluorescent lesions. (C) Macular OCT showing decreased retinal thickness (yellow asterisk), absence of foveal excavation. Macular thickness map with average thickness in RE 214 ± 19 microns and LE 216 ± 16 microns exhibits significant retinal atrophy.

At age of 6 years old, the first evaluation in the ophthalmology department revealed visual acuity as low as 20/800 in her right eye (RE) and 20/50 in her left eye (LE), associated with nyctalopia and fine horizontal nystagmus. Fundoscopy revealed an optic nerve with temporal pallor, macula with scattered pigment clumps, and diffuse chorioretinal atrophy extending to the peripheral retina, along with thinned retinal vessels and pigmentary deposits in a bone‐spicule pattern. Macular OCT showed decreased retinal thickness and absence of foveal excavation (Figure 1). Visual field testing by confrontation detected gross peripheral vision defects in both eyes. Electroretinography and automated visual field testing were not reliable at the age at which they were performed, and it was not possible to repeat. During the 8‐year follow‐up, the best corrected visual acuity decreased to counting fingers in both eyes.

She was referred to the endocrinology department at 10 years and 7 months due to growth retardation and short stature. Her height was 125 cm (−1.88 SDS), her weight 35 kg (0.01 SDS), her body mass index (BMI) 22.05 (1.66 SDS), and her target height 161.3 cm (+0.09 SDS) based on Argentinian girls [18]. Bone age was 8 years and 7 months. Further physical examination revealed a high forehead, bushy eyebrows, long and thick eyelashes (trichomegaly), a short neck, and backward‐rotated ears. Her hands had short fourth metacarpals and mild clinodactyly of both fifth fingers.

Developmental milestones were appropriate for age, and the neurological examination was unremarkable, except for nystagmus secondary to low vision and a mild neurodevelopmental delay, predominantly characterized by learning difficulties.

She was diagnosed with growth hormone deficiency, confirmed by a peak GH of 2 ng/mL on two arginine/clonidine stimulation tests (reference: > 4.7 ng/mL) [19], and central hypothyroidism, evidenced by an inappropriately normal TSH of 2.46 mU/L (reference: 0.5–5 mU/L) in the setting of low free thyroxine of 0.77 µg/dL (reference: < 0.88 µg/dL). The ACTH–cortisol axis was normal, and brain MRI was unremarkable, including a normal appearance of the hypothalamic–pituitary region, with no structural abnormalities detected.

She started hormone replacement therapy: levothyroxine and recombinant growth hormone (rGH) with a good response, reaching her target height 164 cm (0.54 SDS) at 17 years old. At age 14 years old, she was diagnosed with hypogonadotropic hypogonadism and began treatment with estrogen to induce puberty (Figure S1). At present, she is on combined oral contraceptives. Although she required continuous academic support due to both cognitive impairment and progressive visual loss, she was able to successfully complete high school.

### 3.2. Biallelic *PNPLA6* Variants

**Figure 2:**
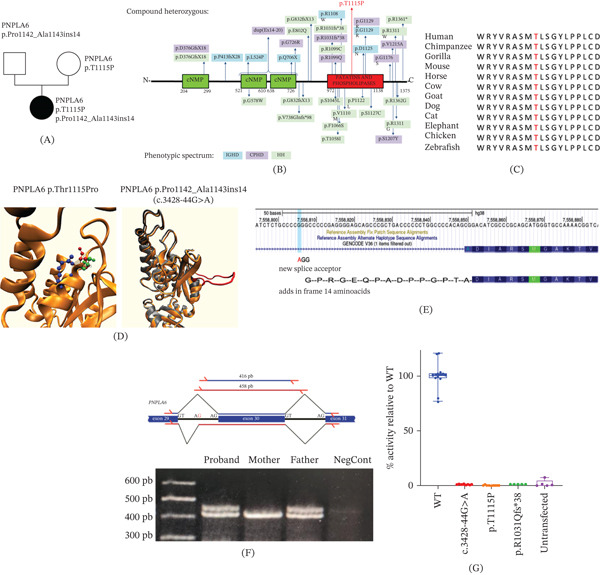
Biallelic genetic variants in *PNPLA6*. (A) Pedigree showing inheritance of p.T1115P from the mother and c.3428‐44G>A from the father. (B) PNPLA6 protein structure and variants related to IGHD or CPHD and other associated phenotypes. The missense variant from our patient is shown in red. (C) Amino acid conservation of the PNPLA6 sequence between species. (D) PNPLA6 protein structure cartoon showing both variants. Left panel: The mutant proline located at the end of the alpha helix, shown in red, is predicted to destabilize the structure due to its rigidity, in addition to the loss of the interaction between the original Threonine 1115 (green) and the adjacent Arginine 1111 (blue). Right panel: The original structure is shown in gray, while the mutant structure is shown in orange. A loop that interrupts an alpha‐helix of the catalytic domain is highlighted in red. (E) Integrated viewer showing splice variant, in frame the extra 14 amino acids. (F) Confirmation of splicing variant in proband and father by RT‐PCR. Primer design scheme and expected sizes when amplifying the WT mRNA (blue) or the variant (red) (upper panel). Agarose gel of the PCR from cDNA samples. Lanes from left to right: 100 bp ladder, proband, mother, father, and negative PCR control (no CDNA template) (lower panel). (G) Both *PNPLA6* variants exhibit loss of enzymatic activity. The activity of neuropathy target esterase (NTE) was defined as the difference in phenyl valerate hydrolysis in the presence of paraoxon (which inhibits basal esterase activity) and the combination of paraoxon and mipafox (which inhibits both basal esterase activity and NTE). Finally, absorbance was measured at 486 nm (*n* = 5, *n* for each experimental condition represents the number of independent samples measured).

We used panel sequencing based on smMIPS and found a missense variant *PNPLA6:* c.3343A>C, p.Thr1115Pro in this patient (Table 1) [9]. This variant was also present in the healthy mother. All cases attributed to variants in *PNPLA6* are homozygous or compound heterozygous. Based on this, we decided to perform WGS in the trio, including the proband, mother, and father. We found a second variant in *PNPLA6:* c.3428‐44G>A p.Pro1142_Ala1143ins14, which is predicted to generate a novel splice acceptor site (SpliceAI score 0.98), resulting in the insertion of 14 amino acids in‐frame the PNPLA6 protein (Figure 2). This variant was also present in the healthy father and absent in the mother. The intronic variant has a minor allele frequency of 1.5e − 5 in gnomAD with no reports of homozygous individuals, while the missense variant is absent in the database; neither variant has been reported on ClinVar. Position 1115 in the protein is highly conserved from humans to zebrafish in all the homologous proteins tested, implying a higher probability of pathogenicity (Figure 2C). The amino acid change p.Thr1115Pro is potentially highly disruptive, as proline usually produces stiff turns that can alter protein structure (Figure 2D and Table 1). Located within the catalytic domain, this substitution results in a predicted *Δ* *Δ* *G* of −2.6 kcal/mol according to DDMut, suggesting a significant reduction in the stability of the protein’s native conformation of the mutant compared to the wild type [20]. We did not find any additional variants of concern in this patient.

**Table 1:** Variants found in the patient.

Gene (transcript)	cDNA position	Predicted protein change	ACMG criteria	Classification
*PNPLA6* (NM_001166114.2)	c.3313A>C	p.T1115P	PM1, PM2, PM3, PP2, PP3	LP
	c.3428‐44G>A	p.Pro1142_Ala1143ins14	PS3, PM2, PM3	LP

### 3.3. Functional Assays

To assess whether the c.3428‐44G>A variant affected splicing, we used RT‐PCR to amplify *PNPLA6* transcripts from peripheral blood obtained from the father, mother, and proband. Abnormal splicing was observed in the sample from the patient and father, but not in the mother (Figure 2F).

In order to elucidate whether the p.Thr1115Pro and p.Pro1142_Ala1143ins14 variants are loss of function, we performed NTE enzymatic assays using an established protocol [3]. Wild type and mutant proteins were expressed at similar levels after measuring the total quantity of NTE‐specific protein using previously defined methods [15] (Table S1 and Figure S2). Both the missense variant and the splicing variant completely abolish NTE activity (Figure 2G). Thus, the compound heterozygous variants are pathogenic and could explain the patient phenotype.

### 3.4. PNPLA6 Is Expressed in the Developing Pituitary and Is Transiently Coexpressed With the Stem Cell Marker SOX2

**Figure 3:**
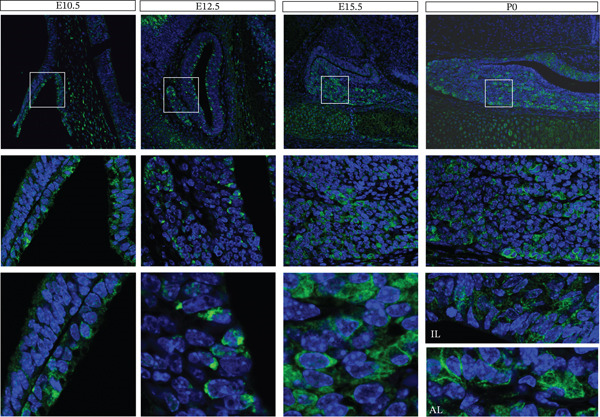
PNPLA6 is expressed in early mouse pituitary development. Sagittal sections of mouse embryos collected at e10.5, 12.5, and e15.5 and at birth (p0) were stained with DAPI to visualize nuclei and with antibodies to PNPLA6 (green). The positions of the anterior lobe (AL), intermediate lobe (IL), and posterior lobe (PL) are indicated. Upper panel 20× and middle/lower panels 63×.

**Figure 4:**
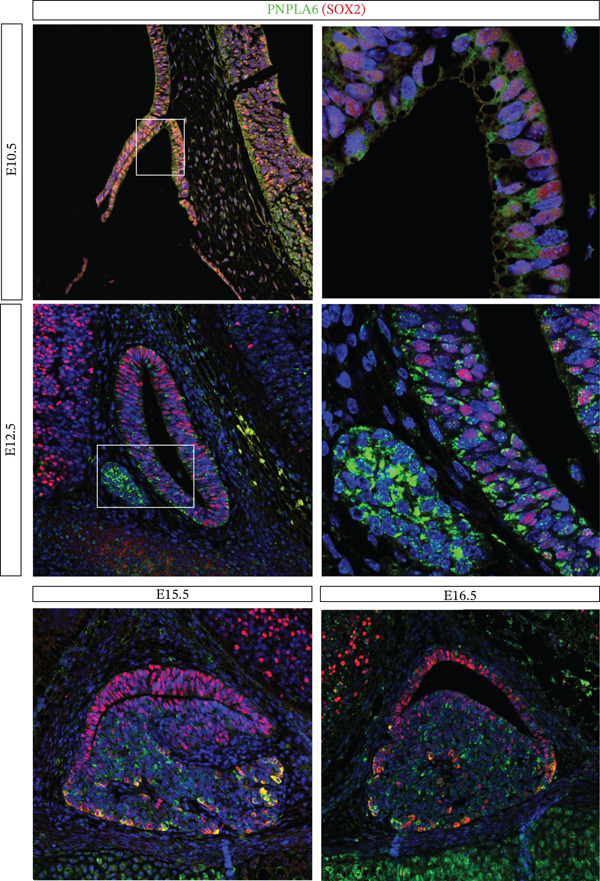
PNPLA6 is expressed in the stem cell niche. Coimmunostaining sections from e10.5, e12.5, e15.5, and e16.6 embryos with SOX2 and PNPLA6 reveal colocalization of these proteins in stem cells with SOX2 staining the nucleus in red and PNPLA6 staining the cytoplasm in green. Pictures are 20× and 63×.

To understand the potential role of PNPLA6 in pituitary development and function, PNPLA6 expression was assessed in mouse embryos and P0 pituitaries using immunohistochemical staining. First, PNPLA6 expression was noted throughout the pituitary primordium at e10.5 (Figure 3). Coimmunostaining with the stem cell marker SOX2 confirmed that PNPLA6 is expressed in pituitary stem cells (Figure 4). Later, at e12.5, PNPLA6 expression is decreased in the dorsal region of the Rathke’s pouch, where the highly proliferative SOX2 positive cells still predominate, and it is highly expressed in the rostral, ventral aspect of the pituitary, where cells are beginning to differentiate (Figures 3 and 4). At e15.5 and e16.5, PNPLA6‐expressing cells do not colocalize with SOX2 (Figure 4). Instead, PNPLA6 is expressed in the parenchyma of the anterior lobe, where cells are differentiating into hormone‐producing cells. Thus, PNPLA6 is initially expressed in SOX2+ stem cells in the embryonic pituitary, and the double staining of PNPLA6 with SOX2 is transient, ceasing as cells migrate from the stem cell niche and differentiate.

### 3.5. PNPLA6 Expression Predominates in Somatotropes and Corticotropes in the Anterior and Intermediate Postnatal Pituitary Gland

**Figure 5:**
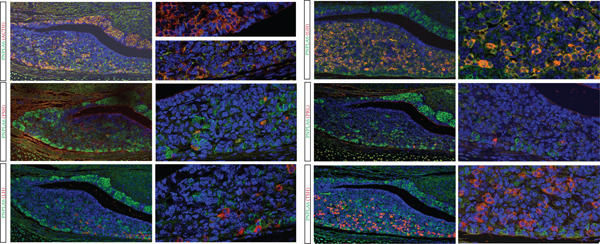
PNPLA6 is expressed in GH and ACTH‐producing cells in mouse pituitaries at birth. Coronal sections of pituitaries from newborn mice (p0) were costained with PNPLA6 antibodies (green) and antibodies for individual pituitary hormones (red): GH, PRL, TSH, ACTH, FSH, and LH. Pictures are 20× and 63×.

We determined the cell‐specific expression of PNPLA6 in the pituitary gland at P0 using immunohistochemistry to costain for PNPLA6 and each of the pituitary hormones individually. The majority of PNPLA6 expressing cells were positive for GH and POMC. We did not detect any colocalization of PNPLA6 and LH*β*, FSH*β*, or PRL at P0. Very few cells coexpressed PNPLA6 and TSH at P0 (Figure 5). It is intriguing the high level of PNPLA6 expression in the POMC‐expressing cells of the intermediate lobe, where the protein is cleaved to make melanocyte‐stimulating hormone, and in the anterior lobe, where it is processed to produce ACTH. Similar observations were present in adult pituitaries, except for some gonadotroph cells expressing PNPLA6 (Figure S3).

## 4. Discussion

Our findings expand the phenotypic spectrum associated with compound heterozygous loss‐of‐function variants in *PNPLA6*. Previous reports of *PNPLA6*‐related CPHD have described individuals with small anterior pituitaries, GH deficiency, and variable involvement of TSH and gonadotropins. The phenotype in our patient—marked GH deficiency with TSH and gonadotropin deficiencies—aligns closely with these earlier cases, further supporting a role for *PNPLA6* in anterior pituitary development and function.

Expression data provide insight into the underlying mechanism. *PNPLA6* is expressed in anterior pituitary stem cells during embryogenesis in mice (e10.5–e15.5) and in hormone‐producing cells. *PNPLA6* is found in most GH‐ and ACTH‐producing cells in newborn mice and remains expressed in adult GH cells. In human embryos, *PNPLA6* transcripts were detected at Carnegie Stage 23, which is roughly equivalent to e13.5 in mice. Expression was detected in the parenchyma and stem cell zone, consistent with our mouse results [1, 4]. This cell‐specific expression pattern suggests that early loss of *PNPLA6* function could disrupt stem cell maintenance or differentiation, leading to pituitary hypoplasia, while later expression in hormone‐producing cells may be critical for their long‐term survival or activity.

Our patient present vision loss. Retinal involvement has been observed in other *PNPLA6*‐associated syndromes, such as Oliver–McFarlane and Boucher–Neuhäuser syndromes. In a recent study, the authors showed that PNPLA6 plays an essential role in retinal homeostasis by controlling choline availability for phospholipid recycling [7].

The variability in gonadotropin and TSH deficiencies among reported patients hints at tissue‐specific sensitivities or compensatory mechanisms. Interestingly, the mechanism of GH deficiency may differ from that of luteinizing hormone (LH) or TSH deficiency [21] reflecting distinct developmental windows or requirements for *PNPLA6* activity across pituitary lineages.

The apparent discrepancy between PNPLA6’s predominant postnatal expression in somatotropes and corticotropes and the broader hormonal deficiencies observed in our patient, affecting GH, TSH, and gonadotropins, can be reconciled by several nonmutually exclusive mechanisms. First, PNPLA6 is broadly expressed in SOX2+ pituitary stem cells as early as e10.5 mice, prior to lineage specification. Loss of function at this early stage could impair the maintenance or differentiation of multiple progenitor populations, independent of the postnatal expression pattern. Second, different pituitary cell lineages may exhibit varying thresholds of sensitivity to ER phospholipid dysregulation. Third, it is important to acknowledge that the expression data presented here were obtained in the mouse pituitary, and direct extrapolation to human pituitary biology should be made with caution. Species differences in PNPLA6 expression patterns may contribute to the discordance observed between the mouse expression data and the clinical phenotype of our patient, as has been documented for other pituitary developmental regulators [22]. Together, these considerations highlight that postnatal expression patterns alone are insufficient to predict which hormonal axes will be clinically affected and underscore the importance of early developmental expression in stem cell populations as well as the inherent limitations of cross‐species comparisons. Additional functional studies, particularly in human pituitary organoid or stem cell models, could help clarify whether *PNPLA6* loss primarily affects progenitor cell maintenance, terminal differentiation, or postnatal hormone production.

Overall, our report underscores *PNPLA6* as a critical factor in anterior pituitary development and expands the catalog of *PNPLA6* variants, and associated phenotypes will further illuminate genotype–phenotype correlations and refine clinical management strategies.

## Author Contributions

Conceptualization: S.V. and M.I.P‐M. Patient recruitment and clinical data: E.V., M.C., M.I.D.P., A.B., and S.H.V. Process samples and data analysis: S.V., J.L., M.A.C., J.M.M., L.I.G., M.B., R.B.H., and M.M. Writing and editing: S.A.C. and M.I.P‐M.

## Funding

The study was funded by 3billion, NIH (R01HD108156), and Agencia Nacional de Promoción Científica y Tecnológica (10.13039/501100003074, PICT APLICADO 2021).

## Disclosure

All authors read and approved the final manuscript.

## Ethics Statement

The study was conducted in accordance with the Declaration of Helsinki and approved by the ethical committee of the hospital. Informed consent forms were signed by either adult individuals with congenital hypopituitarism or the parents/guardians of the children with congenital hypopituitarism.

## Consent

Any information detailed in this manuscript was covered by the informed consent forms that were signed by either adult individuals with congenital hypopituitarism or the parents/guardians of the children with congenital hypopituitarism.

## Conflicts of Interest

The authors declare no conflicts of interest.

## Supporting Information

Additional supporting information can be found online in the Supporting Information section.

## Supporting information


**Supporting Information 1** Table S1: Coomassie blue quantification (control relative to Figure 2G).

**Supporting Information 2** Figure S1: Growth chart showing the initiation of hormone replacement therapies. T4, levothyroxine, dose 2 *μ*g/kg/day; rhGH, recombinant human growth hormone dose 0.26 mg/kg/week; E2, estrogen (17*β*‐estradiol), dose 0.28 mg/day.

**Supporting Information 3** Figure S2: NTE expression level between conditions (control experiment for Figure 2G).

**Supporting Information 4** Figure S3: PNPLA6 is expressed in some FSH*β* cells in adult pituitaries. Three‐month‐old mouse pituitaries were stained for PNPLA6 in red and FSH*β* in green. Pictures are 40×.

## Data Availability

The data that support the findings of this study are available from the corresponding author upon reasonable request.
