# Supplementary material for: Compound Heterozygous Variants in the Phospholipase Gene PNPLA6 Cause Hypopituitarism and Vision Loss
Source: Hum Mutat. 2026 Jun 19;2026:4515038. doi: 10.1155/humu/4515038 (PMC13282554; doi:10.1155/humu/4515038)
Supplement: Supplementary file 2 — Supporting Information 2 Figure S1: Growth chart showing the initiation of hormone replacement therapies. T4, levothyroxine, dose 2 μg/kg/day; rhGH, recombinant human growth hormone dose 0.26 mg/kg/week; E2, estrogen (17β‐estradiol), dose 0.28 mg/day. [file HUMU-2026-4515038-s001.pdf]

Figure S1: Growth chart showing the initiation of hormone replacement therapies

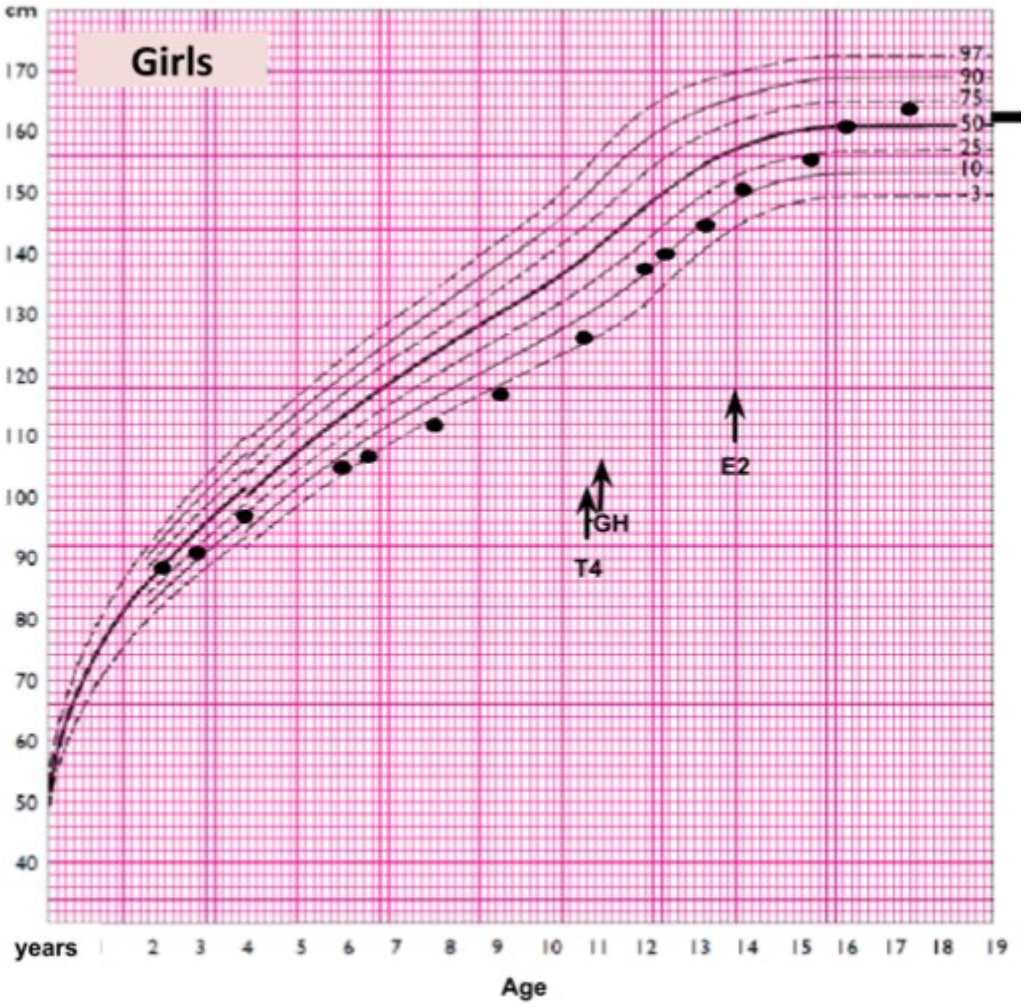

T4: levothyroxine, dose 2 µg/kg/day, rhGH: recombinant human growth hormone dose 0,26mg/kg/week, E2: estrogen (17β- estradiol), dose 0,28mg/day.
