# Supplementary figures and images for: Compound Heterozygous Variants in the Phospholipase Gene PNPLA6 Cause Hypopituitarism and Vision Loss
Source: Hum Mutat. 2026 Jun 19;2026:4515038. doi: 10.1155/humu/4515038 (PMC13282554; doi:10.1155/humu/4515038)

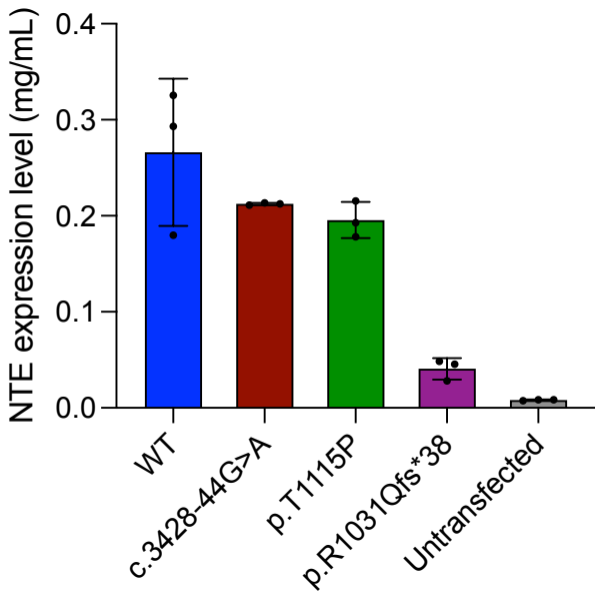

Supplement: Supplementary file 3 — Supporting Information 3 Figure S2: NTE expression level between conditions (control experiment for Figure 2G). [file HUMU-2026-4515038-s004.pdf]

Figure Suple 3

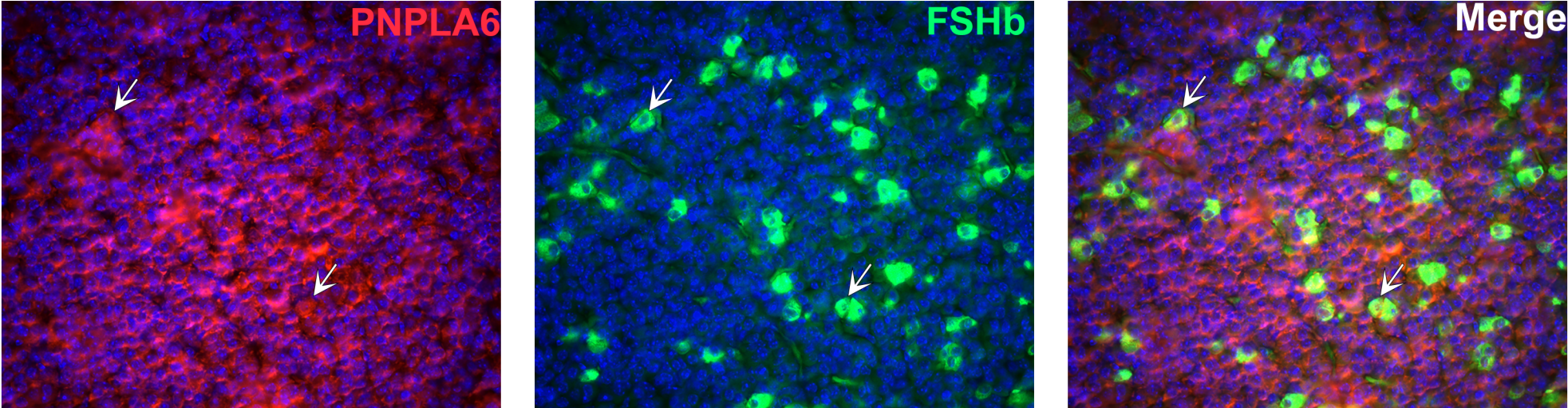

Supplement: Supplementary file 4 — Supporting Information 4 Figure S3: PNPLA6 is expressed in some FSHβ cells in adult pituitaries. Three‐month‐old mouse pituitaries were stained for PNPLA6 in red and FSHβ in green. Pictures are 40×. [file HUMU-2026-4515038-s003.pdf]
